# Lack of Association between rs17568 Polymorphism in OX40 Gene and Myocardial Infarction, Southern of Iran

**DOI:** 10.5539/gjhs.v8n6p41

**Published:** 2015-09-21

**Authors:** Mehrnoosh Maalhagh, Mohammad Shojaei, Saeideh Erfanian, Abdolreza Sotoodeh Jahromi, Mohammad Sadegh Sanie, Alireza Yusefi, Hassan Zabetian, Hossein Hakimelahi, Abdolhossien Madani, Mohammad Hojjat-Farsangi

**Affiliations:** 1 Student Research Committee, Jahrom University of Medical Sciences, Jahrom, Iran; 2 Research Center for Non-Communicable Diseases, Jahrom University of Medical Sciences, Jahrom, Iran; 3 Research Center for Social Determinants in Health Promotion, Hormozgan University of Medical Sciences, Bandarabbas, Iran; 4 Department of Oncology-Pathology, Immune and Gene therapy Lab, Cancer Center Karolinska (CCK), Karolinska University Hospital Solna and Karolinska Institute, Stockholm, Sweden

**Keywords:** premature myocardial infarction, OX40, Gene polymorphism

## Abstract

Tumor necrosis factor (TNF) is one of the inflammatory cytokines which has an important role in inflammation and migration of other inflammatory cells to the atherosclerotic plaques. OX40 is a member of the TNF super family receptor protein. OX40 and OX40 ligand are co-stimulators for T-cells and can increase inflammatory response in atherosclerotic plaques. The aim of this study was to determine the association of rs17568 polymorphism in OX40 gene with premature myocardial infarction. This case control study was done on 100 patients with premature acute myocardial infarction (AMI) and a similar number of sex, age and some other cardiovascular risk factor matched healthy people. The OX40 rs17568 polymorphism was genotyped, using PCR-RFLP method. A-allele frequency of rs17568 SNP was lower non-significantly in Premature AMI, compared to healthy subjects (49% vs. 51%). The analysis of rs17568 (A/G) polymorphism showed an odds ratio of 1.127 (95% CI: 0.635-1.999; P= 0.686) for the GG genotype and 5.761 (95% CI: 1.200-27.655; P= 0.029) for the AG genotype, compared to the AA genotype. The results of this study indicate that the rs17568 SNP of OX40 gene is not associated with premature AMI in the evaluated population.

## 1. Introduction

Atherosclerosis underlies the leading cause of death in industrialized societies and is likely soon to attain this status worldwide (Lloyd-Jones et al., 2010). When coronary artery disease develops, vessels will be obstructed which cause acute coronary syndrome (ACS) (Shea et al., 1984; Thom et al., 2006). ACS has an abrupt onset and presents with a severe chest pain or other equivalent symptoms. ACS divides into three types based on changes in electrocardiogram (ECG) includes unstable angina and non-ST elevation myocardial infarction which have no change in ECG and ST-elevation myocardial infarction that presents with elevation in ST segment of ECG (Khosravi et al., 2008)

Underlying pathology which causes ACS is atherosclerotic plaques that lead to failure of blood supply in coronary arteries. There are different pathways that play roles in atherosclerotic plaque formation. Genetics and environmental factors are important ones such as family history, obesity, hyperlipidemia, diabetes mellitus, inflammation and hypertension (Shea et al., 1984; Godfrey et al., 1994; Thom et al., 2006; Shojaie et al., 2009; Shojaie et al., 2009; Jahromi et al., 2013; Jahromi et al., 2014).

There are several pathways which cause permanent inflammation in active stage of plaque formation. Tumor necrosis factor (TNF) is one of the inflammatory cytokines which has an important role in inflammation and migration of other inflammatory cells in atherosclerotic plaques (Liu et al., 2009).

TNF super family modulate the cell proliferation and differentiation in immune related process and play roles in auto immune disease which progress due to inflammatory cells over activity (Identification of Single Nucleotide Polymorphisms in the Tumor Necrosis Factor (TNF) and TNF Receptor Superfamily in the Korean Population (Cho et al., 2004).

Atherosclerotic plaques formation is based on endothelial dysfunction, inflammation and immunological processes. Hyperchlostrolemia and increasing lipoproteins in intima layer of vessels cause aggregation of inflammatory cells such as macrophages and T lymphocytes (Khosravi et al., 2008; Liu et al., 2009). Inflammation process in atherosclerosis begins by presence of oxidized low density lipoprotein (LDL) lead to Inflammatory T cells migration to plaque then macrophages detect T cells in plaque and inflammation continues (Antunes et al., 2011).

OX40 is known as CD134, a member of the tumor necrosis factor super family receptor protein (Al-Shamkhani et al., 1997). OX40 and its ligand (OX40L) are co stimulator for T lymphocytes and can increase inflammatory response in atherosclerotic plaques (Stüber & Strober, 1996; Wang et al., 2005).

Recent studies in different societies have shown that genetic variant in expression of OX40/OX40 ligand is related to severity of ACS also can present high risk people and may predict the prognosis of ACS in patients (Cha et al., 2003, Croft et al., 2012).

The aim of this study was to determine the association of rs17568 polymorphism in OX40 gene with premature myocardial infarction.

## 2. Materials & Method

This case control study was done on 100 patients with premature acute myocardial infarction (AMI) (under 50 year old patients) who were referred to medical and educational hospitals of Jahrom University of medical sciences, as case group. Two independent cardiologists corroborated the diagnose of coronary artery disease (CAD) according to the World Health Organization criteria for MI such as chest pain, cold sweating, cardiac enzyme elevation in serum and diagnostic change in ECG. Control group included one hundred sex, age and some CAD risk factors matched healthy individuals.

Exclusion criteria in case group were: death during hospital admission, physical or mental impairment that make participants unable to answer the questionnaire, past medical history of hospital admission due to cardiovascular disease (CVD).

This study conforms to the declaration of Helsinki and approved by the ethics research committee of Jahrom University of Medical Sciences. Each participants had consent to partake in study and based on the testimonial they could leave the study.

### 2.1 Blood Samples

Three ml of venous blood taken and collected in tubes containing EDTA as an anticoagulant then stored at -20 °C in order to extract DNA.

### 2.2 Extraction of DNA and PCR

Extraction of DNA was done by kit based on the instruction of the product. The part of GATA2 gene [A transcription factor GATA2 (GATA-binding protein 2), which regulates several endothelial-specific genes, as a novel susceptibility gene for Coronary Heart Disease], including the rs17568 Polymorphism multiplied by polymerase chain reaction (PCR) by master mix tube of Bioneer Company (South Korea). The reaction, containing MgCl2 solution Mμ 1.5, dNTP each 250Mμ, and 0.2 μL (1 unit) Taq polymerase enzyme, DNA template 2.5μL, 10 pmol primers each, and sterile water to 20 μL total reaction volume. PCR was done using thermal cycler device.

PCR cycles were explained as below:

PCR conditions for genotyping using either set of primers included an initial denaturation at 94 °C for 5 minutes followed by 35 cycles and 94 °C for 30 seconds, 55 °C for 30 seconds, 72 °C for 1 minute and finally 72 °C for 10 minutes. Final PCR product was assessed in agarose gel containing Ethidium bromide.

**Table:** 

Primer	Sequence
5’_ CCA GCC ACG CAG CCC CAG AA -3’	rs17568
5’_ CTG GGT GGG GTC CACAGGAGGG -3’	

Specific primers that used are mentioned in table below:

Gene runner software used to determine the duplicated parts of primers. Gene sequences were confirmed by blast program. These primers yielded a PCR product of 188 base pairs (bp) spanning the Mun I (Fermentase Company, concentration: 10 U/ul), polymorphic site. Amplification products were digested with the restrictive enzyme Mun I wild type (genotype AA), the PCR product was digested into DNA fragments of 160 and 28 bp. The mutant G allele did not undergo digestion with the enzyme Mun I. DNA fragments obtained after restrictive enzyme digestion and the DNA size marker were electrophoresed on a 3% gel agarose and stained with ethidium bromide.

### 2.3 Statistical Analysis

Correlation between polymorphisms in OX 40 gene and acute coronary syndrome in the case and the control groups determined with Odds ratio (OR), Chi-square and Fisher exact tests. In deductive part of study the differences in biochemical markers and demographic information evaluated with T test. All analyses were done by SPSS version 11.5.

## 3. Results

There were 200 participants in this study with ages in range of 30-50 years old. Mean of age in case group who developed with MI and referred to medical and educational hospitals of Jahrom University of medical sciences was 41.5±4.9 years and mean of age in control group was 42.5±6.6 years. There was no significant difference between mean of age in the both groups (P=0.197).

There was no significant difference between gender (P=0.876) and smoking (P=0.323) in case and control group. According to the results of study 70% (70 people) of case group had family history of CAD. In control group 90% of participants had no family history of CAD. There was a notable difference between case and control group (P<0.001). In case group 25% of participants had hypertension (HTN), 23% had hyperlipidemia (HLP) and 25% had diabetes mellitus (DM). There were significant differences involving with HTN (0.001), HLP (0.07), and DM (0.010) in case group compared with control group (Table 1).

**Table 1:** Demographic and CVD risk factors for the case and the control groups

Variables	Control group N=100	Case group N=100	P value
Males (%)	30	32	**0.761**
Age (year)	6.6±42.5	4.9±41.5	**0.197**
Smoking (%)	27	25	**0.321**
Diabetes (%)	11	25	**0.010**
Family history (%)	10	70	**0.001**
Hyperlipidemia (%)	9	23	**0.070**
Hypertension (%)	8	25	**0.001**

Frequency of genotype in polymorphism of rs17568 is defined in Table 2.

**Table 2:** Frequency of genotype in rs17568 polymorphism

	Control group N=100	Case group N=100	P value	Odds Ratio (confidence interval)
AA	46 (%46)	53 (%53)	Ref	**Ref**
AG	10 (%10)	2 (%2)	0.029	**5.761 (1.200-27.655)**
GG	44(%44)	45 (%45)	0.686	**1.127 (0.635-1.999)**
AA	46 (%46)	53 (%53)	0.322	**0.433-1.317**
AG+GG	54 (%54)	47 (%47)		
**Allele frequency**				
A	102 (%51)	108 (%49)	0.548	**0.887 (0.599-1.313)**
G	98 (%49)	92 (%46)		

A-allele frequency of rs17568 SNP was lower non-significantly in Premature AMI, compared to healthy subjects (49% vs. 51%). The analysis of rs17568 (A/G) polymorphism showed an odds ratio of 1.127 (95% CI: 0.635-1.999; P= 0.686) for the GG genotype and 5.761 (95% CI: 1.200-27.655; P= 0.029) for the AG genotype, compared to the AA genotype. The results of this study indicate that the rs17568 SNP of OX40 gene is not associated with premature AMI in the evaluated population.

## 4. Discussion

Present study shows that CVD risk factors such as hypertension, family history and diabetes play important role in early occurrence of CVD. Therefore family history and life style play essential and obvious role in CAD.

There are several studies in field of OX40 gene polymorphism that has controversial results. Wang X and et al study done in 2005 proved that rs17568 polymorphism in OX40 gene effect on serum level of HDL (Wang et al., 2005). Another study demonstrates that there is a reciprocal effect between rs17568 variant and ACS occurrence (Andreassi et al., 2012).

There are studies that show controversial results in association between rs3850641 polymorphism in tumor necrosis factor super family 4 and occurrence of myocardial infarction (MI) (Matijevic et al., 2011; Andreassi et al., 2012; Vecoli et al., 2014).

Croft M, et al and two other studies revealed that there is a significant correlation between rs3850641 polymorphism and MI (Cha et al., 2003; Matijevic et al., 2011; Croft et al., 2012).

On the other hand in 3 individual studies done there wasn’t significant association between rs3850641 polymorphism and outbreaks of ACS (Koch et al., 2008; Chen et al., 2011; Cheng et al., 2011).

In studies mentioned the average sample sizes were 150 individuals and unpaired t test, ANOVA and Pearson 2-way test and Chi-square was done. Fisher exact tests

According to the results of studies mentioned above in some polymorphisms there are significant correlation with occurrence of MI. In present study there was no significant correlation between rs17568 polymorphism and MI incidence.

Rui Liu and et al study demonstrated the association between rs17568 polymorphism and lipid profile (Liu et al., 2013). Huang Q and et al couldn’t reveal the role of rs17568 polymorphism in atherosclerotic cerebral infarction (Huang et al., 2014). Results of these studies may clarify the role of rs17568 polymorphism in lipid profile changes more than occurrence of MI.

Compared with other studies present study couldn’t find obvious correlation between rs17568 polymorphism and premature MI occurrence but revealed the role of family history and CVD risk factors in premature MI.
